# Ultrasound measurement of thyroid volume in euthyroid children under 3 years of age

**DOI:** 10.1590/0100-3984.2020.0016

**Published:** 2021

**Authors:** Luís Ronan Marquez Ferreira de Souza, Nathalie de Almeida Sedassari, Eduarda Lemes Dias, Fernanda Cristina Mattos Dib, Heloisa Marcelina Cunha Palhares, Adriana Paula da Silva, Janaíne Machado Tomé, Maria de Fátima Borges

**Affiliations:** 1 Universidade Federal do Triângulo Mineiro (UFTM), Uberaba, MG, Brazil.

**Keywords:** Thyroid diseases, Congenital hypothyroidism, Ultrasonography, Child development, Child health, Doenças da glândula tireoide, Hipotireoidismo congênito, Ultrassonografia, Desenvolvimento infantil, Saúde da criança

## Abstract

**Objective:**

To establish ultrasound reference values for thyroid volumes in children up to 3 years of age, given that ultrasound of the thyroid is an essential examination in the diagnosis of childhood thyroid disease.

**Materials and Methods:**

This was a prospective study conducted in an iodine-sufficient city in southeastern Brazil. A total of 100 healthy children underwent clinical evaluation, anthropometric examination, and cervical ultrasound in accordance with conventional protocols. We evaluated characteristics such as echotexture, thyroid lobe volume, and total thyroid volume. The children were divided into five groups, by age: < 2 months; 2-12 months; 12-18 months; 18-24 months; and 24-36 months.

**Results:**

The mean thyroid volume was lower in the < 2 month age group than in the other groups (0.4 mL vs. 0.18-0.70 mL; *p* < 0.001). For the subjects between 2 and 36 months of age, the mean volume was 1.0 mL (range, 0.30-2.0 mL). No other significant differences were observed between groups, thyroid lobes, or gender. However, body mass index correlated significantly with total thyroid volume (*r* = 0.347; *p* = 0.001).

**Conclusion:**

The mean thyroid dimensions were smallest in the < 2 month age group (0.35 ± 0.16 mL). For the subjects between 2 and 36 months of age, a reference value of 0.85 ± 0.42 mL can be used. Our data could guide the diagnostic investigation of thyroid disease, especially congenital hypothyroidism, in childhood.

## Abstract (pt)

**Objetivo:**

Estabelecer valores de referência ultrassonográficos de volumes tireoidianos em crianças de até três anos de idade, por ser exame essencial no diagnóstico de doença tireoidiana infantil.

**Materiais e Métodos:**

Trata-se de estudo prospectivo conduzido em cidade do sudeste brasileiro suficiente em iodo. Participaram 100 crianças saudáveis, submetidas a exame clinicoantropométrico e ultrassonografia cervical seguindo protocolos convencionais. Características como ecotextura e volumes dos lobos e da tireoide total foram obtidos. Segundo a idade, as crianças foram distribuídas em cinco grupos: < 2 meses; 2-12 meses; 12-18 meses; 18-24 meses; e 24-36 meses.

**Resultados:**

No grupo < 2 meses os volumes tireoidianos foram menores (*p* < 0,001) do que nos demais (0,4 mL; 0,18-0,70 mL). Além dessa idade, o volume total médio foi 1,0 mL (0,30-2,0 mL) e não diferiu entre os grupos. Não foram observadas diferenças entre lobos ou relacionadas a gênero. Houve correlação (*r* = 0,347; *p* = 0,001) entre índice de massa corporal e volume total.

**Conclusão:**

As dimensões tireoidianas foram menores até dois meses (0,35 ± 0,16 mL), e a partir dessa idade os mesmos valores de referência podem ser usados até 36 meses (0,85 ± 0,42 mL). Tais dados podem orientar a investigação diagnóstica, especialmente no hipotireoidismo congênito.

## INTRODUCTION

Thyroid diseases are among the most prevalent endocrine disorders in childhood and throughout life, ranging from those occurring at birth (congenital hypothyroidism) to those occurring during adolescence or adulthood (hypothyroidism or hyperthyroidism). In addition, the detection of benign or malignant thyroid nodules may require specialized attention and care; in such cases, thyroid ultrasound plays a key role. Since the 1980s, ultrasound has been widely used in the evaluation of the thyroid, because it is a noninvasive method that is accessible and does not expose patients to radiation, allowing the diagnosis and follow-up of patients with thyroid diseases^(1-4)^. Ultrasound has been considered the main first-line method of image evaluation in congenital hypothyroidism, being a useful tool in the diagnosis of embryonic developmental disorders such as dysgenesis (including ectopia, agenesis, and hypoplasia) and thyroid dyshormonogenesis^(5-8)^.

From a technical and evolutive perspective, thyroid ultrasound enables the detection of ectopic and topical thyroids; precise volume calculation of its lobes and isthmus; and characterization of the parenchyma and its abnormalities. Thyroid volume is influenced by various factors, including age, dietary iodine intake, and anthropometric characteristics^(9,10)^.

Reference values for thyroid volume in euthyroid children have been reported, although most have been for neonates^(11-16)^ or for children ≥ 6 years of age^(17-22)^, thyroid volume having been poorly reported in younger children and infants^(11,23,24)^. Because ultrasound examination is considered an essential element of the initial evaluation of any thyroid disease in childhood, the aim of this study was to develop a protocol for standardizing the measurements of thyroid volume in euthyroid children under 3 years of age in an iodine-sufficient city in southeastern Brazil.

## MATERIALS AND METHODS

The Research Ethics Committee of the Universidade Federal do Triângulo Mineiro (UFTM) approved the study (Reference no. 1496). Written informed consent was obtained from the primary caregiver of each child.

We enrolled a convenience sample of euthyroid children from the Pediatric Outpatient Clinic of the Hospital das Clínicas da UFTM, in the city of Uberaba, located in the southeastern region of Brazil, which, according to some studies^(25,26)^ it is a region sufficient in dietary iodine. The inclusion criteria were being ≥ 5 days of age, being healthy on clinical examination, and having had a normal neonatal heel prick test (Guthrie test) result. Children who had previously been diagnosed with thyroid disease were excluded, as were those who were overweight/obese and those who had been born prematurely or small for gestational age. Physical examinations were performed in order to collect anthropometric data-weight, length, height, body mass index (BMI), and body surface area (BSA)-in accordance with the standards established by the World Health Organization^(27)^.

**Figure 1:**
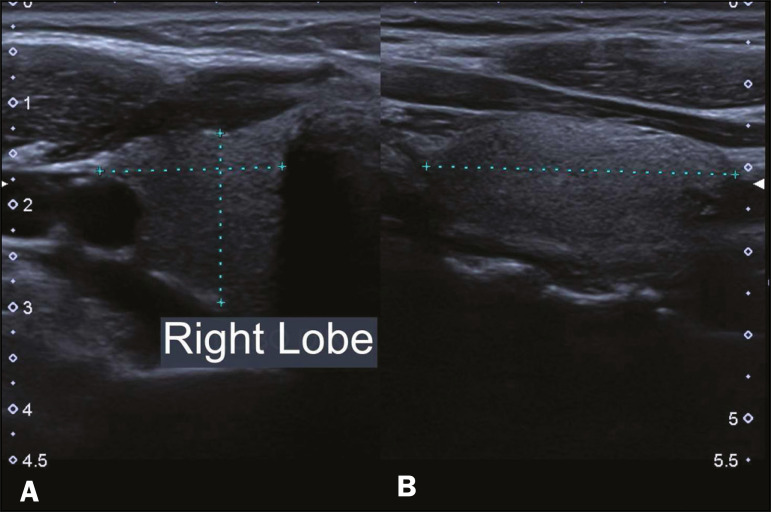
Thyroid ultrasound examination of a euthyroid child. The right thyroid lobe (between the markers) is observed in the transverse section for laterolateral measurement (A) and in the longitudinal section for craniocaudal and anteroposterior measurements (B).

**Figure 2:**
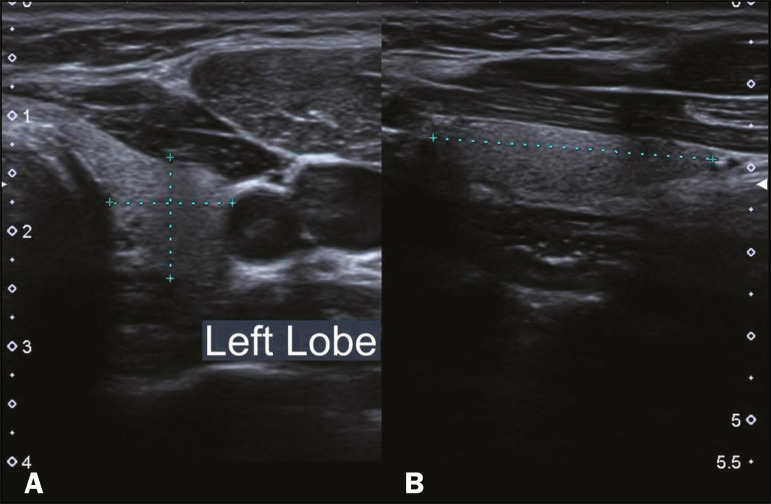
Thyroid ultrasound examination of a euthyroid child. The left thyroid lobe (between the markers) is observed in the transverse section for laterolateral measurement (A) and in the longitudinal section for craniocaudal measurement (B).

The study sample comprised 100 children (64 males and 36 females), who were initially divided into six groups, by age: < 2 months; 2-6 months; 6-12 months; 12-18 months; 18-24 months; and 24-36 months. All children underwent ultrasound of the thyroid to assess the characteristics of the gland, including its position, volume, texture, and other aspects (Figures 1 and 2). The examinations were performed with the patients in the supine position, with extension of the neck to facilitate the anatomical analysis^(9,10)^. In the initial evaluation of the thyroid, its lobes and isthmus were characterized, as were its position in the neck, echotexture, dimensions, and volume. The thyroid volume was calculated according to the formula π/σ×depth×length×width. The measurements were taken in the longitudinal plane (craniocaudal and anteroposterior dimensions). Total thyroid volume was determined by adding the volumes of both lobes, the isthmus being disregarded because it is very small at these ages. Lesions in the thyroid parenchyma were recorded separately^(9,10)^.

All ultrasound scans were performed with the same ultrasound system (Aplio 400; Canon Medical Systems, Tustin, CA, USA). The examinations were performed by a radiologist and recorded in the Digital Imaging and Communications in Medicine format. The images were subsequently analyzed by another radiologist with considerable experience in thyroid ultrasound.

### Statistical analysis

For each age group, thyroid volumes are expressed as mean ± standard deviation and range. The right thyroid lobe volume, left thyroid lobe volume, and total gland volume were evaluated separately.

The distribution of the variables was determined with the Kolmogorov-Smirnov test, and those that did not meet the assumptions of normality underwent log, log(X + 1), square-root, or square-root(X + 0.5) transformation. For comparisons between three or more groups, we used analysis of variance followed by the Tukey's post hoc test. Pearson's correlation coefficient was used in order to quantify correlations between the variables BMI, BSA, and thyroid volume. To compare the right and left thyroid volumes, Student's t-test was used.

The data obtained were double-entered into a Microsoft Excel spreadsheet. All statistical analyses were performed with the IBM SPSS Statistics software package, version 21.0 (IBM Corp., Armonk, NY, USA). The level of significance adopted for all tests was 5% (*p* ≤ 0.05).

## RESULTS

In the initial comparative analysis, we found no significant differences between the 2-6 month and 6-12 month age groups in terms of the mean values for right thyroid lobe volume, left thyroid lobe volume, and total thyroid volume (*p* = 0.999). Therefore, those two groups were merged into a single group for analysis. Thus, the six original age groups became five: < 2 months (n = 33); 2-12 months (n = 19); 12-18 months (n = 15); 18-24 months (n = 13); and 24-36 months (n = 20).

Table 1 shows the mean right thyroid lobe volume, left thyroid lobe volume, and total thyroid volume for all five age groups, those values being lowest (0.18 ± 0.08 mL, 0.17 ± 0.08 mL, and 0.35 ± 0.16 mL, respectively) in the < 2 month age group. Thyroid volumes did not differ significantly among the other groups. In addition to the data for each group, mean values were calculated collectively for the children 2-36 months of age. There were no significant differences between the right and left thyroid lobe volumes in any of the age groups evaluated (*p* > 0.05 for all).

**Table 1:** Thyroid volumes obtained by ultrasound in euthyroid children, by age group.

Age group	N	Thyroid volume					
		Right lobe (mL)			Left lobe (mL)		Total (mL)
		Mean ± S	D (range)		Mean ± SD (range)		Mean ± SD (range)
0-2 months	33	0.18 ± 0.08	(0.08-0.34)*		0.17 ± 0.08 (0.06-0.40)*		0.35 ± 0.16 (0.18-0.70)*
2-12 months	19	0.44 ± 0.25	(0.10-1.00)		0.45 ± 0.24 (0.10-1.10)		0.90 ± 0.50 (0.30-2.10)
12-18 months	15	0.56 ± 0.35	(0.10-1.30)		0.44 ± 0.23 (0.12-0.90)		1.00 ± 0.58 (0.30-1.90)
18-24 months	13	0.37 ± 0.20	(0.12-0.72)		0.40 ± 0.20 (0.19-0.83)		0.77 ± 0.40 (0.31-1.55)
24-36 months	20	0.40 ± 0.10	(0.12-0.60)		0.38 ± 0.20 (0.14-0.75)		0.78 ± 0.30 (0.35-1.29)
2-36 months	67	0.43 ± 0.24	(0.10-1.30)		0.42 ± 0.20 (0.10-1.10)		0.85 ± 0.42 (0.30-2.10)

* *p* < 0.001 vs. the 2-12 month, 12-18 month, 18-24 month, and 24-36 month age groups (analysis of variance).

In the sample as a whole, thyroid volume showed a weak but significant correlation with BMI (*r* = 0.343; *p* < 0.001), although it did not correlate with BSA (Table 2). When we stratified the subjects by gender, we found significant correlations between BMI and total volume in females (*r* = 0.326; *p* = 0.043) and males (*r* = 0.370; *p* = 0.03). However, thyroid volume did not correlate with BMI or BSA in any of the individual age groups.

**Table 2:** Correlations of total thyroid volume with BMI and BSA in euthyroid children, by age group.

Age group	N	Total thyroid volume vs. BMI			Total thyroid volume vs. BSA	
		*r*	*P*		*r*	*P*
0-2 months	33	0.290	0.102		0.152	0.398
2-12 months	19	0.359	0.131		0.180	0.460
12-18 months	15	-0.314	0.296		-0.454	0.119
18-24 months	13	0.174	0.569		0.120	0.697
24-36 months	20	-0.119	0.617		0.002	0.993
Sample as a whole	100	0.343	0.001*		0.106	0.300

* Pearson's correlation coefficient.

## DISCUSSION

Screening programs employing the neonatal heel prick test (Guthrie test) have shown a relevant prevalence of congenital hypothyroidism in various populations^(28)^. The search for simple, effective diagnostic methods that facilitate the definition of the etiology of thyroid disease is justified by these important findings^(6)^. The estimated incidence of congenital hypothyroidism among Hispanics and Native Americans in the United States is 1:2,000 live births, similar to the 1:2,017 live births reported in studies conducted in Brazil^(28,29)^. As previously mentioned, thyroid ultrasound is a noninvasive method, is easy to perform, and is widely available, which makes it a viable auxiliary method for the early diagnosis of thyroid diseases. It enables us to distinguish between normal and abnormal structures in the thyroid, as well as to identify dysgenesis (ectopia, agenesis, and hypoplasia). If necessary in order to clarify the etiology of congenital hypothyroidism, it is possible to select specific cases to undergo further tests, such as molecular tests and other imaging examinations^(9,10)^.

In the present study, we standardized reference values for thyroid volumes in children from day 5 of life to 3 years of age, with the objective of providing data that would be useful in the diagnosis and follow-up of children with congenital hypothyroidism, given that neonatal screening for congenital hypothyroidism occurs in the first month of life and that the etiological investigation is performed at approximately 3 years of age^(29)^. Around this age, the treatment must be discontinued in order to perform thyroid scintigraphy and specific tests. In 3-year-olds, ultrasound evaluation of the thyroid, in conjunction with molecular tests for congenital thyroid defects could preclude the need for costly investigations that would require the treatment to be interrupted^(9,10,29)^.

We determined the mean total thyroid volume for each age group on the basis of the hypothesis that the volume would increase with age, as suggested in the literature^(17-23)^. In the present study, thyroid volumes were lowest in the < 2 month age group (i.e., neonates), although they did not differ significantly among the other age groups. The mean total thyroid volume in the < 2 month age group (0.35 ± 0.16 mL) is even lower than that reported in the literature for neonates^(11-16)^. That could be explained by the fact that most such studies have been conducted in areas of iodine deficiency, which could lead to a compensatory (adaptive) increase in thyroid volume. The present study involved children from an area of southeastern Brazil that is currently considered iodine sufficient^(25,26)^. Such differences also indicate the need for regional determination of thyroid volumes.

Most studies reporting age-related increases in thyroid volume involved children ≥ 6 years of age. In fact, as children approach puberty they tend to accumulate fat, and the hormonal changes during puberty also change the overall composition of the body, including that of the thyroid^(21-24)^.

There have been few studies evaluating the thyroid volumes of children in the age groups analyzed in the present study. In a study conducted in the Istanbul region of Turkey, Aydıner et al.^(23)^ evaluated 422 subjects 0-55 years of age, by age group. Although their age stratification was slightly different, the authors reported reference ranges for total thyroid volume of 0.37-1.23 mL in the 0- to 1-year age group and 0.28-2.14 mL in the 1- to 3-year age group, which are similar to those found in this study. The authors also stated that there have been iodine supplementation programs in their country for the last 15 years, underscoring the need to update the reference values for thyroid volumes in the various age groups. In another study, conducted in Fukushima, Japan, an iodine-sufficient area, by Suzuki et al.^(24)^ evaluated individuals 0-19 years of age. They stratified patients from year to year and by gender, as well as evaluating the 0- to 3-year age group separately. The reference ranges for the right thyroid lobe, left thyroid lobe, and total thyroid volumes were 0.2-1.5 mL, 0.2-1.4 mL, and 0.3-1.7 mL, respectively, similar to ours.

Some authors have reported that, in addition to regional differences related to dietary iodine content, ethnicity, and age, thyroid volumes correlate with weight, height, and BSA^(17-23)^. In the present study, we detected a significant correlation between BMI and total thyroid volume when we analyzed the sample as a whole and when we stratified the subjects by gender. However, we found no such correlation within the individual age groups, which was attributed to the small sample size. We found no correlation between thyroid volume and BSA, although (as expected) there was a significant correlation between BMI and BSA.

In a study involving subjects 8-15 years of age, Moradi et al.^(30)^ found that thyroid volume correlated significantly with BMI in boys but not in girls. That difference was probably due to the inclusion of adolescents in their sample, given that body composition differs between teenager boys and girls.

Our study has some limitations. Primarily, the sample size was relatively small, given the difficulty in recruiting healthy children in such a young age group. Although that limited our ability to perform intragroup analyses, the number of subjects was sufficient to allow the statistical analysis.

Reference values for thyroid volume in euthyroid children, especially those in the younger age groups, have rarely been reported in the literature. Such values constitute essential data in the initial evaluation of any thyroid disease, especially congenital hypothyroidism. Therefore, the importance of this study is that we present the mean total thyroid volume, as measured by ultrasound, for infants under 2 months of age-0.35 mL (range, 0.18-0.70 mL)-and for individuals between 2 months and 3 years of age-1.0 mL (range, 0.30-2.0 mL).
